# Quantile estimation of semiparametric model with time-varying coefficients for panel count data

**DOI:** 10.1371/journal.pone.0261224

**Published:** 2021-12-13

**Authors:** Yijun Wang, Weiwei Wang

**Affiliations:** 1 School of Statistics and Mathematics, Zhejiang Gongshang University, Hangzhou, Zhejiang Province, China; 2 Collaborative Innovation Center of Statistical Data Engineering, Technology & Application, Zhejiang Gongshang University, Hangzhou, Zhejiang Province, China; Tongji University, CHINA

## Abstract

Panel count data frequently occurs in follow-up studies, such as medical research, social sciences, reliability studies, and tumorigenicity experiences. This type data has been extensively studied by various statistical models with time-invariant regression coefficients. However, the assumption of invariant coefficients may be violated in some reality, and the temporal covariate effects would be of great interest in research studies. This motivates us to consider a more flexible time-varying coefficient model. For statistical inference of the unknown functions, the quantile regression approach based on the B-spline approximation is developed. Asymptotic results on the convergence of the estimators are provided. Some simulation studies are presented to assess the finite-sample performance of the estimators. Finally, two applications of bladder cancer data and US flight delay data are analyzed by the proposed method.

## Introduction

In longitudinal follow-up studies, panel count data is frequently encountered in many fields such as medical research, social sciences, reliability studies, and tumorigenicity experiences, which has been widely analyzed by many authors. This type data is usually collected from the discrete observations in recurrent event process, as the continuous observations might be too expensive to be carried out. Thus, we can only obtain the cumulative occurrence numbers of the events of interest at these discrete observation times.

For the analysis of panel count data, [1, 2] developed the regression analysis approaches to the panel count data model. [3] studied the clustered mixed nonhomogeneous Poisson models of panel count data. [4] considered the spline-based likelihood estimation of the proportional mean model. To describe the potential correlations of the recurrent event process, [5–7] developed some joint models of panel count data by employing some frailty parameters to discuss these correlations. Recently, semiparametric transformation models with informative observation times were studied by many authors, such as [8–10]. More comprehensive introductions about this type data can be referred to the book of [11].

In general, the existing approaches in modeling panel count data are based on the time-invariant coefficients assumption, but which may be violated in practice. In some applications, coefficients may be time-varying, and sometimes it is more vital to detect the temporal impacts on the recurrent event process. For example, in medical studies, we are interested in detecting the temporal impacts of one new drug. Recently, [12, 13] proposed the varying coefficient models for recurrent events. However, the analysis of panel count data with varying coefficients is very limited. Most recently, [14] proposed a partially varying coefficient model of panel count data to account for the nonlinear interactions between covariates. [15] proposed a nonparametric proportional mean model of the panel count data with time-varying coefficients.

Quantile regression is widely used in the analysis of longitudinal data. It can provide more information about the distribution shape of the response and can be used to measure the effect of variables under different percentiles of the distribution. However, quantile regression methodologies for the panel count data are lagging. As the discreteness of the panel count data, quantile regression cannot be directly used. For the first, a smoothing technique (“jitter”) is used for this type data, then the quantile regression can be applied to the smooth data.

In this paper, a semiparametric time-varying coefficient model is formulated. For the inference of the unknown functions, quantile regression method is used for the panel count data, with the unknown functions approximated by the B-spline basis functions. Furthermore, the asymptotic results on the convergence of the estimators are established as well. The main contribution of the paper is that we propose a new spline-based quantile estimation procedure for the time-varying coefficient panel count data model, which has not been discussed in the literature.

## Model specification

Suppose that *n* independent subjects are observed over time. *N*_*i*_(*t*) denotes the cumulative total number of recurrent event occurring at or before time *t* for subject *i*. $\tilde{H_{i}}\left(t\right)$ is a counting process with jumps at the discrete observation times, *t*_*i*,1_ < *t*_*i*,2_ < ⋯. We assume that *t* is in a fix interval ℜ of finite length. Besides, two follow-up times are existed: the potential censoring time $C_{i}^{*}$ and the observation endpoint *T*_*i*_. Thus, only $C_{i}=\text{min}\left(C_{i}^{*},T_{i}\right)$ can be observed in the process, with $\delta_{i}=I\left(C_{i}=C_{i}^{*}\right)$. $C_{i}^{*}$ is assumed to be independent with *N*_*i*_(*t*) and $\tilde{H_{i}}\left(t\right)$. Let $H_{i}\left(t\right)={\tilde{H}}_{i}\left\{\text{min}\left(t,C_{i}\right)\right\}$ denote the real observation process of subject *i*, and $m_{i}={\tilde{H}}_{i}\left(C_{i}\right)$, *i* = 1, ⋯, *n*. Then, *N*_*i*_(*t*) can be only acquired at the time points where *H*_*i*_(*t*) jumps. The total number of the observations is defined as $m=\sum_{i=1}^{n}m_{i}$. Let *Z*_*i*_ be a *p* × 1 vector of covariates. So we can have the independent and identically distributed dataset {*H*_*i*_(*t*), *N*_*i*_(*t*)*dH*_*i*_(*t*), *C*_*i*_, *δ*_*i*_, *Z*_*i*_;*t* ≥ 0, *i* = 1, ⋯, *n*}.

To describe the possible time-varying effects of covariates on *N*_*i*_(*t*), the time-varying coefficient model is proposed as follows.

- (1) Given *Z*_*i*_, the conditional mean function of *N*_*i*_(*t*) is
  $$\begin{array}{c} E\{N_{i}\left(t\right)|Z_{i}\}=\int_{0}^{t}\lambda_{0}\left(u\right)\text{exp}\{{\beta (u)}^{⊤}Z_{i}\}du, \end{array}$$ (1)
  where λ_0_(*u*) is an unspecified smooth baseline intensity function, and *β*(*u*) is an unknown *p* × 1 vector of time-varying regression coefficients.
- (2) Conditional on *Z*_*i*_, $\left\{C_{i},N_{i}\left(t\right),{\tilde{H}}_{i}\left(t\right)\right\}$ are mutually independent.

For the model defined above, [15] developed the likelihood and pseudo-likelihood methods to get the estimation of the baseline intensity function λ_0_(*u*) and the varying coefficient functions *β*(*u*) based on the Poisson distribution assumption on *N*_*i*_(*t*). However, no distribution assumption is specified in this paper and the existed methods cannot be used. In the next section, the spline-based quantile regression is proposed to acquire the estimation of the unknown functions. In the first step, the unknown baseline intensity function and the coefficients are approximated by B-splines. And then, the discrete panel count data become continuous by a smoothing technique. Quantile regression is developed for the inference in the last step.

## Estimation procedure

For the inference of Eq (1), the model can be rewritten as,

$$\begin{array}{c} E\{N_{i}\left(t\right)|X_{i}\}=\int_{0}^{t}\text{exp}\{X_{i}^{⊤}\eta \left(u\right)\}du, \end{array}$$

where $X_{i}={\left(Z_{i}^{⊤},1\right)}^{⊤}$, *η*(*u*) = (*β*(*u*)^⊤^, log{λ_0_(*u*)})^⊤^.

### Approximations of baseline and varying coefficients

Similar as [16], we use the basis expansion method to get the estimation of the unknown functions in this paper. Suppose *η*_*k*_(*u*), *k* = 1, 2, ⋯, *p* + 1, can be approximated by a basis expansion, that is

$$\begin{array}{c} \eta_{k}\left(u\right)\approx \sum_{l=1}^{L_{k}}\gamma_{kl}B_{kl}\left(u\right)=B_{k}{\left(u\right)}^{⊤}\gamma_{k}, \end{array}$$

where $B_{k}\left(u\right)={\left\{B_{k1}\left(u\right),\ldots ,B_{kL_{k}}\left(u\right)\right\}}^{⊤}$ are basis functions, $\gamma_{k}={\left(\gamma_{k1},\ldots ,\gamma_{kL_{k}}\right)}^{⊤}$ and *L*_*k*_ is the number of basis functions. Various basis functions can be used in the expansion such as Fourier basis functions, polynomial basis functions and B-spline functions. In this paper, the B-spline basis is selected in the estimation procedure for calculation simplicity.

The tuning parameter *L*_*k*_ is selected by *L*_*k*_ = *n*_*k*_ + *q*_*k*_ + 1, where *n*_*k*_ is the number of interior knots and *q*_*k*_ is the degree of the B-spline functions. The interior knots of the splines are equally spaced or placed on the sample quantiles of the data in all simulations and applications. The tuning parameter *L*_*k*_ may be different for different *k*. In this paper, we assume that *L*_*k*_ = *L* and *q*_*k*_ = *q* for all *η*_*k*_(*u*). Thus, we define *B*_*k*_(*u*) = *B*(*u*) for simplicity presentation.

### Quantile regression

As quantile regression is a good alternative to the conditional mean models, the quantile regression is considered for the panel count data model. However, quantile regression cannot be directly used as the discreteness of the data *N*_*i*_(*t*). According to the method developed in [17], the “jitter” method is applied to construct continuous random variables. By adding *U*_*ij*_, which is generated from a [0, 1) uniform distribution, we can have

$$\begin{array}{c} N_{i}^{*}\left(t_{ij}\right)=N_{i}\left(t_{ij}\right)+U_{ij}, \end{array}$$

where the noise *U*_*ij*_ is independent of *N*_*i*_(*t*_*ij*_) and *Z*_*i*_. The uniform distribution is used because it allows computational simplifications. The uniform noise, however, is by no means a necessity to jitter the data. The noise may be generated by any continuous distribution with support on [0, 1). Thus, we can get the continuous data $N_{i}^{*}\left(t_{ij}\right)$ and there exists a one-to-one link between the quantiles of *N*_*i*_(*t*_*ij*_) and $N_{i}^{*}\left(t_{ij}\right)$. The regression model of $N_{i}^{*}$ can be written as

$$\begin{array}{c} N_{i}^{*}\left(t_{ij}\right)=\int_{0}^{t}\text{exp}\{X_{i}^{⊤}\eta \left(u\right)\}du+\epsilon_{ij}, \end{array}$$

where *ϵ*_*ij*_ are assumed to be independent of *t*_*ij*_ with unknown cumulative distribution function (cdf) *G*(⋅) and density function *g*(⋅). Besides, the *τ*-th conditional quantile of *ϵ*_*ij*_ is *b*_*τ*_.

The quantile regression loss function is defined as *ρ*_*τ*_(*u*) = *u*[*τ* − *I*(*u* < 0)], *τ* ∈ (0, 1). Then the quantile regression is applied on the smooth data $N_{i}^{*}\left(t_{ij}\right)$ to obtain the estimation of the unknown parameters. Thus, the unknown parameters *ϕ* = (*γ*^⊤^, *b*_*τ*_)^⊤^ can be estimated by minimizing the following objective function Ψ(*ϕ*), that is

$$\begin{array}{c} \Psi \left(\phi \right)=\sum_{i=1}^{n}\sum_{j=1}^{m_{i}}\rho_{\tau }\{N_{i}\left(t_{ij}\right)-\int_{0}^{t_{ij}}\text{exp}\{W{\left(u,X_{i}\right)}^{⊤}\gamma \}du-b_{\tau }\}, \end{array}$$

where *W*(*u*, *X*_*i*_) = *I*_*p*+1_ ⊗ *B*(*u*) ⋅ *X*_*i*_ and $\gamma ={\left(\gamma_{1}^{⊤},\ldots ,\gamma_{p+1}^{⊤}\right)}^{⊤}$.

For the ease of calculation, Gauss-Legendre formula is used to approximate the integral. Thus, we have

$$\begin{array}{c} \int_{0}^{t_{ij}}\text{exp}\{W{\left(u,X_{i}\right)}^{⊤}\gamma \}du\approx \frac{t_{ij}}{2}\sum_{s=1}^{S}\omega_{s}\;\text{exp}[W{\left\{\frac{t_{ij}}{2}\left(1+\Delta_{s}\right),X_{i}\right\}}^{⊤}\gamma ], \end{array}$$

where *ω*_*s*_ is the Gauss coefficient, *S* is the number of the Gauss points and Δ_*s*_ is the Gauss point. The Gauss-Legendre approximation of the objective function Ψ(*ϕ*) can be defined as

$$\begin{array}{c} \Psi \left(\phi \right)\approx \sum_{i=1}^{n}\sum_{j=1}^{m_{i}}\rho_{\tau }\{N_{i}\left(t_{ij}\right)-\frac{t_{ij}}{2}\sum_{s=1}^{S}\omega_{s}\text{exp}[W{\left\{\frac{t_{ij}}{2}\left(1+\Delta_{s}\right),X_{i}\right\}}^{⊤}\gamma ]-b_{\tau }\}. \end{array}$$

Define $\hat{\phi }={\left({\hat{\gamma }}^{⊤},{\hat{b}}_{\tau }\right)}^{⊤}$ be the minimizers of the approximation of the objective function Ψ(*ϕ*). It is nature to get the estimation of the varying coefficient *β*_*k*_(*u*), *k* = 1, ⋯, *p*,

$$\begin{array}{c} {\hat{\beta }}_{k}\left(u\right)\approx \sum_{l=1}^{L}{\hat{\gamma }}_{kl}B_{l}\left(u\right)=B{\left(u\right)}^{⊤}{\hat{\gamma }}_{k}, \end{array}$$

and the baseline intensity function of λ_0_(*u*) can be obtained by

$$\begin{array}{c} {\hat{\lambda }}_{0}\left(u\right)\approx \text{exp}\{\sum_{l=1}^{L}{\hat{\gamma }}_{p+1,l}B_{l}\left(u\right)\}=\text{exp}\{B{\left(u\right)}^{⊤}{\hat{\gamma }}_{p+1}\}. \end{array}$$

Next, we discuss how to select the tuning parameter *L* and the Gauss point number *S*. As proposed by [16], we use the leave-one-subject-out cross-validation (CV) to choose *L* and *S*. Let ${\hat{\gamma }}^{\left(-i\right)}$ and ${\hat{b}}_{\tau }^{\left(-i\right)}$ denote the estimators from the data with the i-th subject deleted. So the leave-one-subject-out CV can be written as

$$\begin{array}{c} CV\left(L,S\right)=\sum_{i=1}^{n}\sum_{j=1}^{m_{i}}\rho_{\tau }\{N_{i}^{*}\left(t_{ij}\right)-\frac{t_{ij}}{2}\sum_{s=1}^{S}\omega_{s}\text{exp}[W{\left\{\frac{t_{ij}}{2}\left(1+\Delta_{s}\right),X_{i}\right\}}^{⊤}\gamma^{\left(-i\right)}]-b_{\tau }^{\left(-i\right)}\}. \end{array}$$

Thus, the tuning parameter *L* and *S* can be selected as

$$\begin{array}{c} \left(L_{CV},S_{CV}\right)=\underset{L,S}{\text{min}}CV\left(L,S\right). \end{array}$$

**Remark 1** *The number L*_*k*_ *of the basis expansion of β*_*k*_ *may be different from each other. However, we assume L*_*k*_ = *L for all k*, *for simplicity*.

## Asymptotic results

The asymptotic results are concluded in this section. Before presenting the results, some regularity conditions are introduced for the first.

(C1) *Z*_*i*_ is uniformly bounded.
(C2) The observation number *m*_*i*_ is bounded by a constant.
(C3) λ_0_(*u*) and *β*_*k*_(*u*), *k* = 1, ⋯, *p*, are *l*-th differentiable and bounded.
(C4) There exists an open subset Ω ⊂ *R*^*pL*+1^, which contains the true parameter ϕ*. The second derivative matrix ∇^2^ *h*(*t*_*ij*_, *X*_*i*_;*ϕ*) of *h*(*t*_*ij*_, *X*_*i*_;*ϕ*) with respect to *ϕ*, satisfies
  $$\begin{array}{c} ∥\nabla^{2}h\left(t_{ij},X_{i};\phi_{1}\right)-\nabla^{2}h\left(t_{ij},X_{i};\phi_{2}\right)∥\leq M_{1}\left(t_{ij},X_{i}\right)∥\phi_{1}-\phi_{2}∥, \end{array}$$
  $$\begin{array}{c} |\frac{\partial^{2}h\left(t_{ij},X_{i};\phi \right)}{\partial \phi_{j}\partial \phi_{k}}|\leq M_{2jk}\left(t_{ij},Z_{i}\right), \end{array}$$
  for all *ϕ* ∈ Ω, with $E[M_{1}^{2}\left(t_{ij},X_{i}\right)]<\infty$, $E[M_{2jk}^{2}\left(t_{ij},X_{i}\right)]<\infty$ for all *j*, *k*.
(C5) $Var(\nabla h_{ij}^{*})=M>0$, $E\{{\left(\nabla h_{ij}^{*}\right)}^{⊗2}\}=\Gamma$, and 0 < *d*_1_ < λ_min_(Γ) ≤ λ_max_(Γ) < *d*_2_ < ∞, where λ_min_(Γ) and λ_max_(Γ) denote the smallest and the largest eigenvalues of Γ.
(C6) *ϵ*_*ij*_ is independent with unknown distribution function *G*(⋅) and density *g*(⋅). Besides, the *τ*-th conditional quantile of *ϵ*_*ij*_ is *ℓ*_*τ*_.

Under these above regularity conditions, the asymptotic results on the convergence of the estimators are displayed in the following theory. For the need of the proofs, a lemma of spline function of [18] is presented. First, define

$$\begin{array}{c} S_{kn}=\{\eta_{kn}:\eta_{kn}=\sum_{l=1}^{L}\gamma_{kl}B_{l}\left(u\right),\left(\gamma_{k1},\cdots ,\gamma_{kL}\right)\in R^{L}\}. \end{array}$$

Let *S*_*kn*_ be the space of splines of degree *q* consisting of functions *η*_*kn*_ satisfying: (i) the function *η*_*kn*_ to each subinterval is a polynomial spline of degree *q*; (ii) for *q* ≥ 1 and 0 ≤ *q*′ ≤ *q*, *η*_*kn*_ is *q*′ times continuously differentiable on the support. Besides, *η*_*k*_ is assumed to satisfy the following regularity condition. Let *l*_1_ ∈ [0, *q*] be a nonnegative integer. The *l*_1_-th derivative, denoted as $\eta_{k}^{\left(l_{1}\right)}$, exists and satisfies the Lipschitz condition of order *v* ∈ (0, 1] such that *ρ* = *l*_1_ + *v* > 0.5 and $|\eta_{k}^{\left(l_{1}\right)}\left(s\right)-\eta_{k}^{\left(l_{1}\right)}\left(t\right)|\leq \delta {\left|s-t\right|}^{v}$, for *s*, *t* ∈ [0, *C*], where *δ* is a positive constant.

**Lemma 1** *There exists η*_*kn*_ ∈ *S*_*kn*_ *such that* ‖*η*_*kn*_ − *η*_*k*_‖_2_ = *O*_*p*_(*L*^−*ρ*^ + *L*^1/2^ *m*^−1/2^). If *L* = *O*{*m*^1/(2*ρ*+1)^}, *then we have* ‖*η*_*kn*_ − *η*_*k*_‖_2_ = *O*_*p*_{(*L*/*m*)^1/2^} = *O*_*p*_{*m*^−*ρ*/(2*ρ*+1)^}.

**Theorem 1** *Suppose the conditions (C1)–(C6) hold and if L* = *O*{*m*^1/(2*ρ*+1)^}, *then we have*

$$\begin{array}{c} \sqrt{m}\left(\hat{\phi }-\phi^{*}\right)\to_{d}N\left\{0,g{\left(b_{\tau }^{*}\right)}^{-2}\tau \left(1-\tau \right)\left(\Gamma^{-1}\right)\right\}. \end{array}$$

*Furthermore, we have*

$$\begin{array}{cc} & {∥{\hat{\beta }}_{k}\left(u\right)-\beta_{k}\left(u\right)∥}_{2}=O_{p}\{{\left(L/m\right)}^{1/2}\},k=1,\cdots ,p,\\ & {∥\;\text{log}{\hat{\lambda }}_{0}\left(u\right)-\;\text{log}\lambda_{0}\left(u\right)∥}_{2}=O_{p}\{{\left(L/m\right)}^{1/2}\}. \end{array}$$

Ignoring the approximation error in the B-spline basis approximation of *β*_*k*_(*u*), *k* = 1, ⋯, *p*, we can have the 100(1 − *α*)% pointwise confidence interval of *β*_*k*_(*u*) under quantile *τ*,

$$\begin{array}{c} {\hat{\beta }}_{k}\left(u\right)\pm z_{2/\alpha }\sqrt{cov\{{\hat{\beta }}_{k}\left(u\right)\}}, \end{array}$$

where *z*_2/*α*_ is the 100(1 − *α*)% percentile of the standard normal distribution and $cov\left\{{\hat{\beta }}_{k}\left(u\right)\right\}=B{\left(u\right)}^{⊤}cov\left({\hat{\gamma }}_{k}\right)B\left(u\right)$. Similar as the baseline function λ_0_(*u*).

## Simulation studies

Three simulation studies are carried out to evaluate the performance of the method developed in this paper. We generated 200 datasets from the time-varying coefficient model, each of size *n* = 100 or 200 independent subjects. For each subject *i*, the endpoint of observation *T*_*i*_ is assumed to be 6 and the censoring time $C_{i}^{*}$ follows the uniform distribution of [*T*_*i*_/2, 3*T*_*i*_/2]. The number of observation times *m*_*i*_ is generated from a discrete uniform distribution {1, 2, 3, 4, 5}. And the observed event times, $\left\{t_{i1},\ldots ,t_{im_{i}}\right\}$, are the order statistics of a random sample size *m*_*i*_ from the uniform distribution over (0, *C*_*i*_). Given *m*_*i*_ and $\left\{t_{i1},\ldots ,t_{im_{i}}\right\}$, the panel count data *N*_*i*_(*t*_*ij*_) can be obtained by the following formula

$$\begin{array}{cc} N_{i}\left(t_{ij}\right) & =N_{i}^{*}[\lambda_{N}\left(t_{i1}\right)]+N_{i}^{*}[\lambda_{N}\left(t_{i2}\right)-\lambda_{N}\left(t_{i1}\right)]\\ & +\cdots +N_{i}^{*}[\lambda_{N}\left(t_{ij}\right)-\lambda_{N}\left(t_{ij-1}\right)], \end{array}$$

for *j* = 1, ⋯*m*_*i*_ and *i* = 1, ⋯*n*. $N_{i}^{*}[\lambda_{N}\left(t_{ij}\right)]$ is the random number generated from the Poisson distribution with mean

$$\begin{array}{c} \int_{0}^{t_{ij}}\lambda_{0}\left(u\right)\text{exp}\{\beta {\left(u\right)}^{⊤}Z_{i}\}du. \end{array}$$

The following three cases are considered:

- Case I: *p* = 1 and the covariate *Z*_*i*_ is generated independently from the [0, 1] uniform distribution. The baseline function is taken as λ_0_(*u*) = 2*u* + 1 and the varying coefficient *β*(*u*) = sin(−*πu*/6).
- Case II: *p* = 1 and the covariate *Z*_*i*_ is generated independently from the [0, 1] uniform distribution. The baseline function is taken as λ_0_(*u*) = 2(*u* + *τ*) and the varying coefficient *β*(*u*) = sin(−*τπu*/6).
- Case III: *p* = 2 and the covariates *Z*_*i*_ are generated from the [0, 1]^2^ uniform distribution with correlation *cor*(*Z*_*ik*_, *Z*_*il*_) = 0.5^|*k*−*l*|^. The baseline function is taken as λ_0_(*u*) = 2*u* + 1 and the varying coefficient *β*_1_(*u*) = *sin*(−*πu*/6) and *β*_2_(*u*) = 2*sin*(−*τπu*/6).

To estimate the smooth functions logλ_0_(*u*) and *β*(*u*), the cubic B-spline functions are selected. Under different quantiles *τ* = {0.25, 0.5, 0.75}, the estimations of Case I–III are presented with sample size *n* = 100 or 200 in Tables 1–3, respectively. The results include the average of the absolute bias values based 100 grid points (BIAS), the average of sampling standard errors based 100 grid points (SSE), the average of the bootstrap standard errors based 100 grid points (BSE) and the average of the estimated 95% coverage probabilities based 100 grid points (CP). It can be seen that the estimations are unbiased under different quantiles. The values of SSE and BSE are close and decrease with the increasing sample size *n*. Besides, from the results of CP, we can note that the Gaussian approximation is appropriate for the estimators.

**Table 1:** BIAS, SSE, BSE and CP of the estimated functions in Case I at different *τ*.

*τ*	*n*	Estimated function	BIAS	SSE	BSE	CP
0.25	100	*β*(*t*)	0.0558	0.8329	0.8355	0.9640
		log λ_0_(*t*)	0.1276	0.4150	0.4453	0.9548
	200	*β*(*t*)	0.0319	0.6826	0.5999	0.9355
		log λ_0_(*t*)	0.1044	0.3853	0.3377	0.9470
0.5	100	*β*(*t*)	0.0598	0.4472	0.4184	0.9623
		log λ_0_(*t*)	0.0341	0.2460	0.2264	0.9750
	200	*β*(*t*)	0.0197	0.3905	0.3684	0.9611
		log λ_0_(*t*)	0.0280	0.1886	0.1725	0.9525
0.75	100	*β*(*t*)	0.0789	0.8014	0.9118	0.9701
		log λ_0_(*t*)	0.0751	0.4935	0.4955	0.9640
	200	*β*(*t*)	0.0553	0.6901	0.6785	0.9368
		log λ_0_(*t*)	0.0599	0.4079	0.3808	0.9472

**Table 2:** BIAS, SSE, BSE and CP of the estimated functions in Case II at different *τ*.

*τ*	*n*	Estimated function	BIAS	SSE	BSE	CP
0.25	100	*β*(*t*)	0.0768	0.9830	0.9890	0.9592
		log λ_0_(*t*)	0.1501	0.4924	0.5356	0.9661
	200	*β*(*t*)	0.0214	0.7345	0.6980	0.9365
		log λ_0_(*t*)	0.1099	0.4021	0.3875	0.9480
0.5	100	*β*(*t*)	0.0455	0.7163	0.6937	0.9250
		log λ_0_(*t*)	0.0746	0.4082	0.4277	0.9425
	200	*β*(*t*)	0.0380	0.4600	0.4511	0.9389
		log λ_0_(*t*)	0.0514	0.2633	0.2601	0.9694
0.75	100	*β*(*t*)	0.0552	0.8944	0.8558	0.9697
		log λ_0_(*t*)	0.0722	0.5191	0.4701	0.9406
	200	*β*(*t*)	0.0398	0.6906	0.6721	0.9355
		log λ_0_(*t*)	0.0529	0.3553	0.3284	0.9640

**Table 3:** BIAS, SSE, BSE and CP of the estimated functions in Case III at different *τ*.

*τ*	*n*	Estimated function	BIAS	SSE	BSE	CP
0.25	100	*β*_1_(*t*)	0.0553	1.0039	0.9841	0.9560
		*β*_2_(*t*)	0.1272	0.9849	0.9762	0.9262
		log λ_0_(*t*)	0.1694	0.7364	0.6911	0.9735
	200	*β*_1_(*t*)	0.0404	0.7205	0.6835	0.9436
		*β*_2_(*t*)	0.0795	0.7557	0.7422	0.9677
		log λ_0_(*t*)	0.1271	0.5135	0.4974	0.9595
0.5	100	*β*_1_(*t*)	0.1330	0.8475	0.8618	0.9205
		*β*_2_(*t*)	0.1641	0.9111	0.8852	0.9455
		log λ_0_(*t*)	0.0431	0.5986	0.5748	0.9625
	200	*β*_1_(*t*)	0.0927	0.6934	0.6520	0.9380
		*β*_2_(*t*)	0.0518	0.7482	0.7249	0.9628
		log λ_0_(*t*)	0.0445	0.4225	0.4418	0.9561
0.75	100	*β*_1_(*t*)	0.1235	0.9436	0.8869	0.9256
		*β*_2_(*t*)	0.1282	0.9478	0.9648	0.9460
		log λ_0_(*t*)	0.0695	0.8331	0.8160	0.9335
	200	*β*_1_(*t*)	0.0383	0.7438	0.7409	0.9510
		*β*_2_(*t*)	0.0825	0.8510	0.8325	0.9246
		log λ_0_(*t*)	0.0507	0.5684	0.5348	0.9714

**Fig 1:**
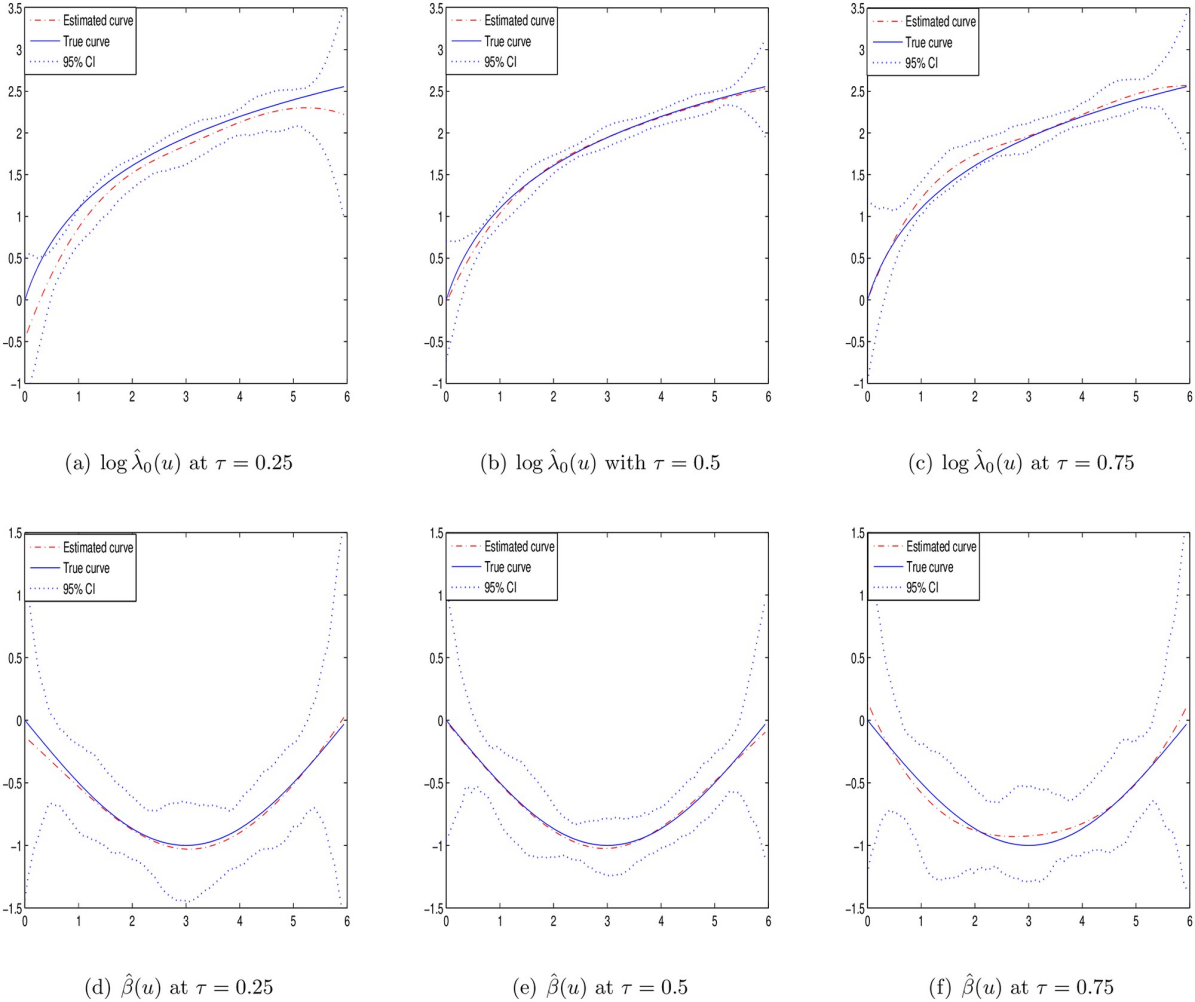
Estimated curves of time-varying functions in case I at different *τ* with *n* = 200.

**Fig 2:**
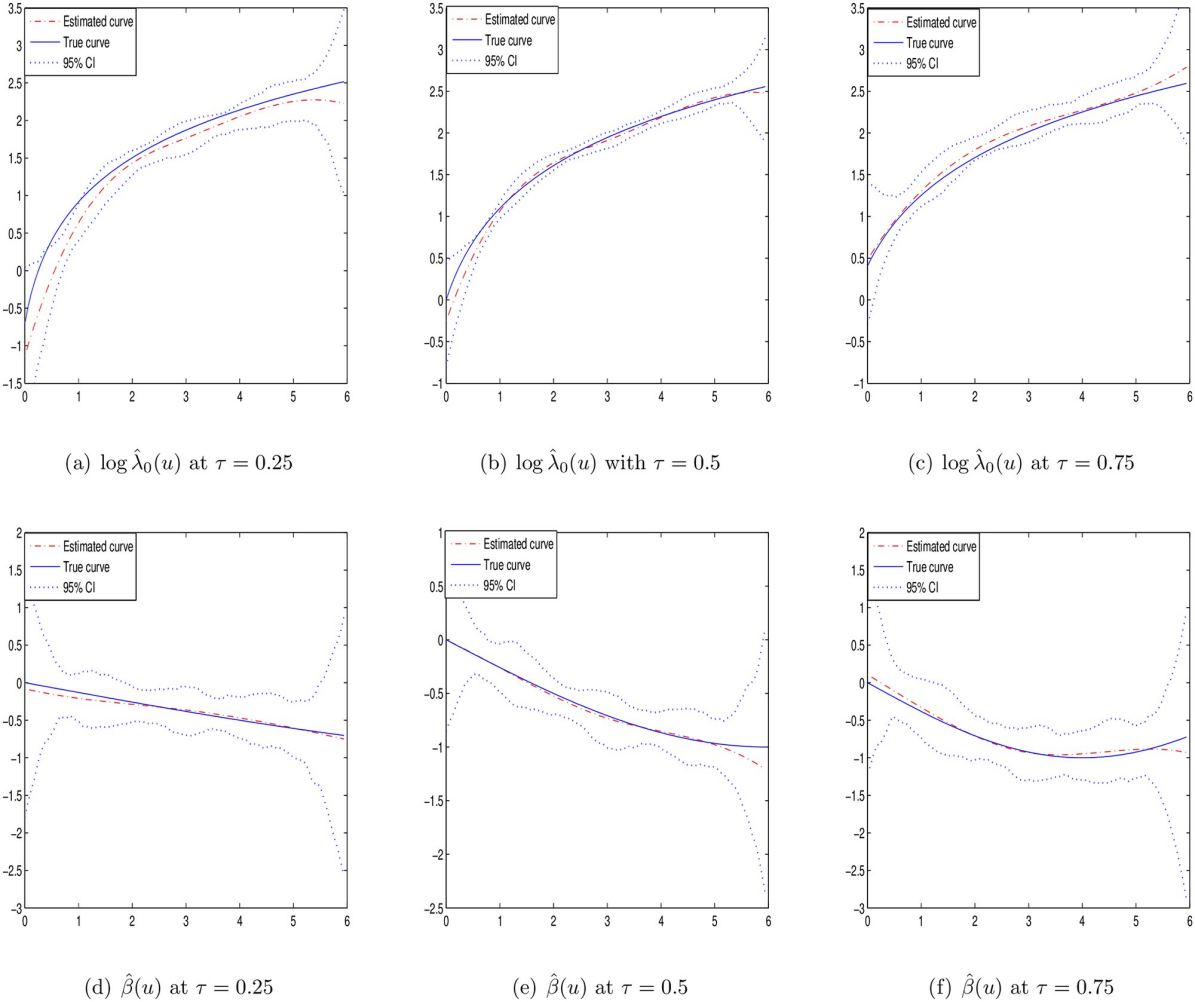
Estimated curves of time-varying functions in case II at different *τ* with *n* = 200.

**Fig 3:**
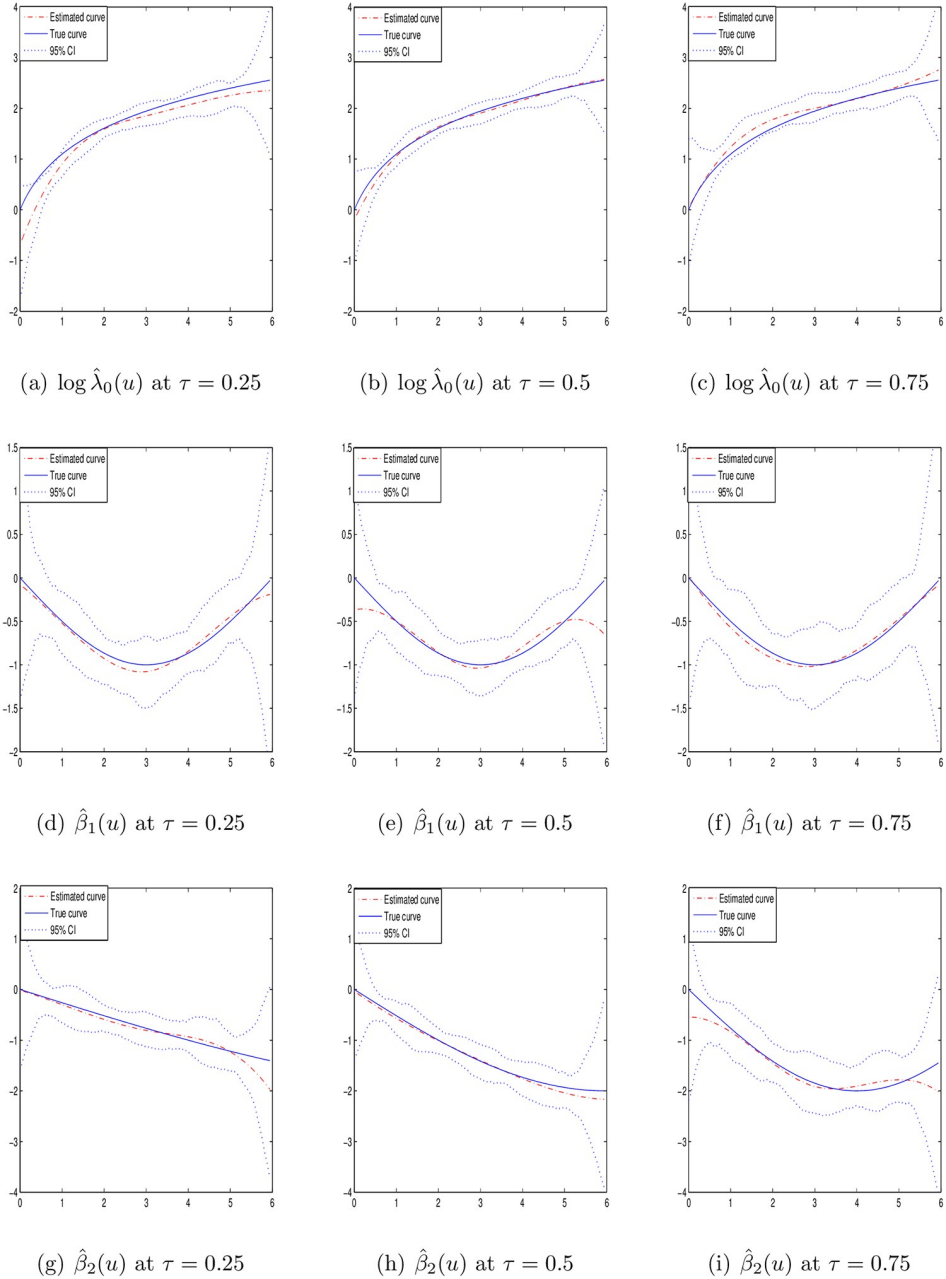
Estimated curves of time-varying functions in case III at different *τ* with *n* = 200.

Figs 1–3 display the estimation curves of the unknown functions log λ_0_(*t*) and *β*(*t*) with *n* = 200. In the figures, the point lines represent the estimated curves, the solid lines represent the true curves and the dotted lines represent the 95% confidence intervals. Based the figures, it is easy to find that the real curves and the estimated curves are very close, which indicates the B-spline estimations of the unknown functions work well. From the simulation results, we note that the estimations under different quantiles are reasonable for log λ_0_(*t*) and *β*(*t*).

## Applications

### Bladder cancer data

Bladder cancer data was collected by the Veterans Administration Cooperative Urological Research Group. In this study, 85 patients were randomly assigned to two treatment groups: placebo group (47) and thiotepa group (38). For each patient, the observation times and the cumulative numbers of the bladder tumors that occurring at or before the observation times are recorded. The observation endpoint is 53 month. What’s more, the initial number of the bladder tumors and the largest initial tumor size for each patient are also recorded. In the literature, the dataset has been discussed by many authors such as [5, 7, 19]. However, time-varying coefficient panel count data model is not considered for this dataset.

In order to describe the temporal impacts of the covariates on the bladder cancer data, the time-varying coefficient model proposed in this paper is applied to these data. For each patient *i*, *N*_*i*_(*t*) is denoted as the cumulative bladder tumors number occurring up to time *t*, and *H*_*i*_(*t*) is denoted as the cumulative observation number up to time t, *i* = 1, ⋯, 85. Furthermore, let *Z*_*i*1_ = 1 if the patient *i* is belonged to the thiotepa group and *Z*_*i*1_ = 0 otherwise. *Z*_*i*2_ is denoted as the initial tumor number and *Z*_*i*3_ is the natural logarithm of the largest initial tumour size plus 1 for each patient *i*. Therefore, we have the model

$$\begin{array}{c} E\{N_{i}\left(t\right)|Z_{i}\}=\int_{0}^{t}\lambda_{0}\left(u\right)\;\text{exp}\;\{\beta_{1}\left(u\right)Z_{i1}+\beta_{2}\left(u\right)Z_{i2}+\beta_{3}\left(u\right)Z_{i3}\}du. \end{array}$$

Then quantile regression estimation is applied to this data. 100 samples are drawn from the data every time and 200 times are repeated in the estimation. Similar to the numerical studies, the unknown functions λ_0_(*t*) and *β*_*k*_(*t*), *k* = 1, 2, 3 are approximated by Cubic B-spline functions. The estimation is implemented under quantiles *τ* ∈ {0.25, 0.5, 0.75}.

**Fig 4:**
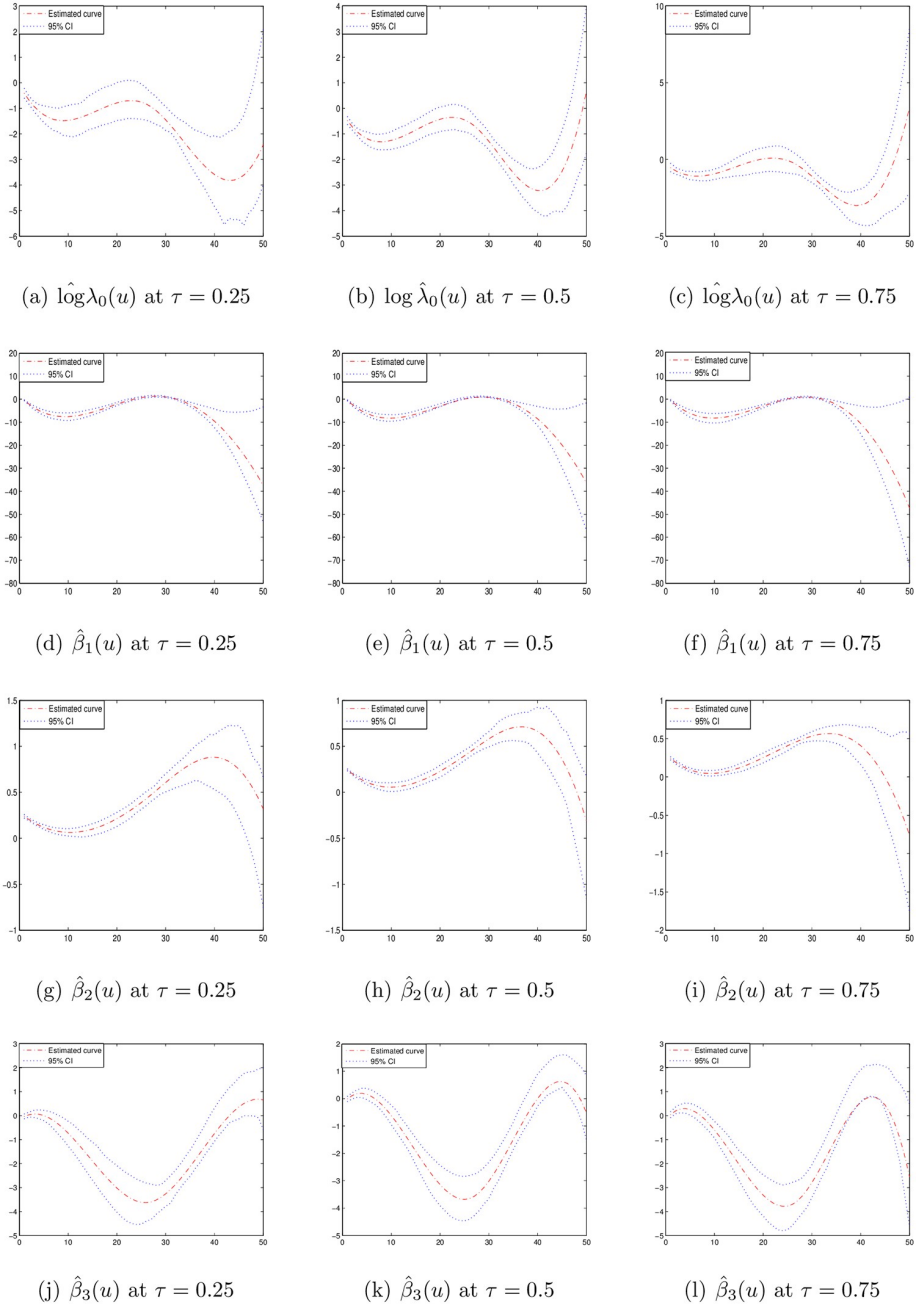
Estimated curves of time-varying functions for bladder cancer data at different *τ*.

The estimation curves of log λ_0_(*t*) and *β*_*k*_(*t*), *k* = 1, 2, 3 are displayed in Fig 4. In general, the thiotepa treatment and the tumor recurrence rate are negatively correlated at different quantiles. Patients in the thiotepa group tend to have less tumor recurrence rate than those in the placebo group. The initial tumor number is positively correlated with the recurrence rate and the largest initial tumor size is negatively correlated with the recurrence rate. These above conclusions are consistent with [19]. Furthermore, we can find the covariates impacts are varying during the observation time and the impacts are different at different quantiles. Thus, more information can be obtained from the quantile regression of the time-varying coefficient panel count data model than the other analysis in the existing literature.

### US flight delay data

In this subsection, 2015 US flight delay data (available from https://www.kaggle.com/usdot/flight-delays) is analyzed with the time-varying coefficient panel count data model. This dataset was collected from the U.S. Department of Transportation’s (DOT) monthly Air Travel Consumer Report. The report contained information about the numbers of on-time, delayed, canceled, and diverted flights. The dataset included 9794 flights which were observed during 3 months in the year of 2015. The numbers of delays for each flight are recorded between the observation times. The observation times of each flight are the same and the observation interval is 7 days. Besides, the average departure delay time and the average flight distance of each flight are also recorded.

In order to describe the temporal covariates impacts on the flight delays, the time-varying coefficient model proposed in this paper is used to these data. For each flight *i*, *N*_*i*_(*t*) is denoted as the cumulative flight delay number that had occurred up to time *t*, *H*_*i*_(*t*) is denoted as the cumulative observation number up to time t, *i* = 1, ⋯, 9794. Furthermore, we define *Z*_*i*1_ as the average time of the departure delay and *Z*_*i*2_ as the average distance of the flight *i*. Therefore, we have the model

$$\begin{array}{c} E\{N_{i}\left(t\right)|Z_{i}\}=\int_{0}^{t}\lambda_{0}\left(u\right)\;\text{exp}\;\{\beta_{1}\left(u\right)Z_{i1}+\beta_{2}\left(u\right)Z_{i2}\}du. \end{array}$$

Then spline-based quantile estimation is applied to this data. Similarly, the unknown functions λ_0_(*t*) and *β*_*k*_(*t*), *k* = 1, 2 are also approximated by Cubic B-spline functions. The estimation is implemented under quantiles *τ* ∈ {0.25, 0.5, 0.75}.

As the sample size of the dataset is large, it is time-consuming or even not possible to read the entire dataset in practice due to the limited memory. Besides, the direct analysis can be infeasible, mainly due to the computing memory or computing time. In order to deal with the massive data, parallel computing method is developed by [20, 21]. In parallel computing method, we split the original dataset into a family of disjoint sub-sample blocks with equal size for the first. More precisely, the data structure can be defined as the following form:

$$\begin{array}{c} S=[S_{k}=\{H_{ki}\left(t\right),N_{ki}\left(t\right)dH_{ki}\left(t\right),Z_{ki};t\geq 0,i=1,\cdots ,m\},k=1,\cdots ,K], \end{array}$$

where the original dataset *S* is of size *n* = *K* × *m* which is partitioned to *K* subsample blocks *S*_*k*_ each consist *m* samples which are randomly picked up from the dataset *S*.

Thus, the estimation procedure proposed can be implemented for every block *S*_*k*_, *k* = 1, ⋯, *K* and the estimated values of unknown parameters for each block *S*_*k*_ is denoted as ${\left\{\hat{\eta }\left(t\right)\right\}}_{k}$. Similar to the method introduced in [21], the final full-sample estimators can be generated by

$$\begin{array}{c} \hat{\eta }\left(t\right)=\sum_{k=1}^{K}{\left\{\hat{\eta }\left(t\right)\right\}}_{k}. \end{array}$$

**Fig 5:**
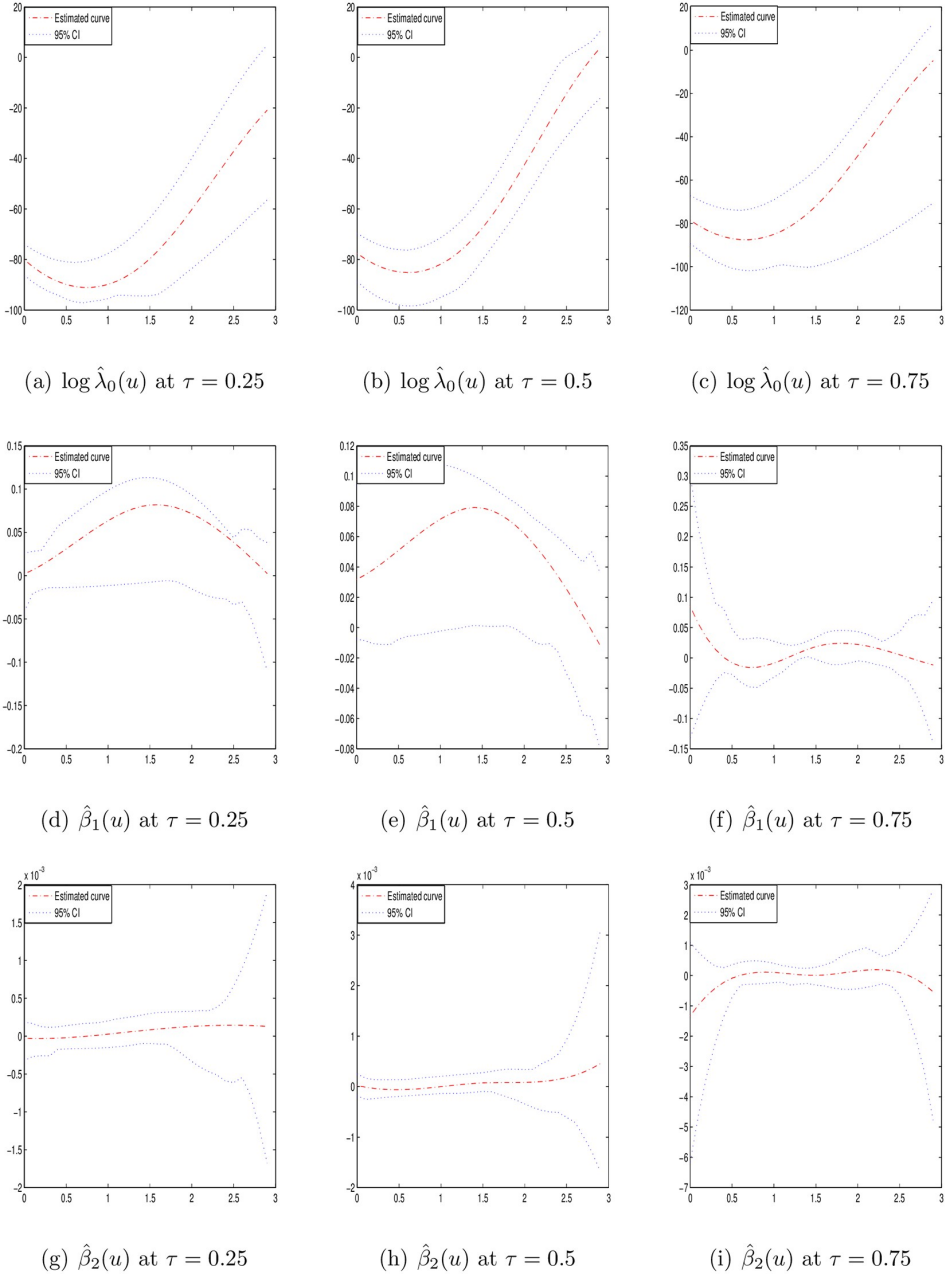
Estimated curves of time-varying functions for US flight delay data at different *τ*.

The estimation curves of log λ_0_(*t*) and *β*_*k*_(*t*), *k* = 1, 2, under different quantiles *τ* ∈ {0.25, 0.5, 0.75} are displayed in Fig 5. From Fig 5, we can find that the departure delay time is positively correlated with the cumulative flight delay numbers. Besides, the impact of the departure delay time is varying over the time under different quantiles and the impact is different at different quantiles. However, the effect of the flight distance is not significant on the flight delay numbers.

## Concluding remarks

In this paper, we proposed a spline-based quantile regression estimation method in the time-varying coefficient panel count data model. This model discussed in our paper is more general than [15], with no Poisson restriction on the recurrent event process. To get the estimations, B-splines are used to approximate the unknown functions log λ_0_(*t*) and *β*(*t*) for the first, and then a smoothing technique is applied to obtain the continuation of the discrete panel count data. Finally, the spline-based quantile regression approach is developed at different quantiles. Some simulations are presented to evaluate the performance of the proposed approach and two applications are analyzed to demonstrate its effectiveness in this paper.

Recently, the Enron e-mail corpus which was a massive set of the e-mail messages, have been discussed by many authors, such as [22]. If we are interested in the number of interactions of all pairs of individuals in this longitudinal observations, as usual in network analysis, the snapshots are applied to model this longitudinal networks, then, this is a standard panel count dataset with massive observations. Furthermore, in this paper, we only considered the situation with low dimensional covariates, which may be not unpracticable in the applications. As the high-dimensional covariates may be existed, variable selection methods can be considered for the time-varying coefficient model. This will be an important topic for our further studies. Besides, reliability data and traffic data have been studied by many authors, such as [23–26]. This will be interesting to study the quantile regression estimation of such data.

## Proof of Theorem 1

Define $\gamma^{*},b_{\tau }^{*}$ as the true but unknown values of $\gamma ,b_{\tau }^{*}$, $u_{1}=\sqrt{m}\left(\gamma -\gamma^{*}\right)$, $u_{2}=\sqrt{m}\left(b_{\tau }-b_{\tau }^{*}\right)$, $u={\left(u_{1}^{⊤},u_{2}\right)}^{⊤}$, *ϕ* = (*γ*^⊤^, *b*_*τ*_)^⊤^ and $h\left(t_{ij},X_{i};\phi \right)=\int_{0}^{t_{ij}}\text{exp}\{W{\left(u,X_{i}\right)}^{⊤}\gamma \}du+b_{\tau }.$

Let

$$\begin{array}{cc} H(\phi^{*}) & =\sum_{i=1}^{n}\sum_{j=1}^{m_{i}}\rho_{\tau }\{N_{i}^{*}\left(t_{ij}\right)-\int_{0}^{t_{ij}}\text{exp}\{W{\left(u,X_{i}\right)}^{⊤}\gamma^{*}\}du-b_{\tau }^{*}\}\\ & =\sum_{i=1}^{n}\sum_{j=1}^{m_{i}}\rho_{\tau }\{\epsilon_{ij}-b_{\tau }^{*}+\int_{0}^{t_{ij}}\text{exp}\{X_{i}^{⊤}\eta \left(u\right)\}du-\int_{0}^{t_{ij}}\text{exp}\{W{\left(u,X_{i}\right)}^{⊤}\gamma^{*}\}du\}\\ & =\sum_{i=1}^{n}\sum_{j=1}^{m_{i}}\rho_{\tau }(\epsilon_{ij}-b_{\tau }^{*}+r_{ij}), \end{array}$$

where

$$\begin{array}{c} r_{ij}=\int_{0}^{t_{ij}}\text{exp}\{X_{i}^{⊤}\eta \left(u\right)\}du-\int_{0}^{t_{ij}}\text{exp}\{W{\left(u,X_{i}\right)}^{⊤}\gamma^{*}\}du. \end{array}$$

By the Taylor expansion, we can have *r*_*ij*_ = *o*_*p*_(1). Besides,

$$\begin{array}{cc} H(\phi^{*}+u/\sqrt{m}) & =\sum_{i=1}^{n}\sum_{j=1}^{m_{i}}\rho_{\tau }\{N_{i}^{*}\left(t_{ij}\right)-h\left(t_{ij},X_{i};\phi^{*}+u/\sqrt{m}\right)\}\\ & =\sum_{i=1}^{n}\sum_{j=1}^{m_{i}}\rho_{\tau }\{\epsilon_{ij}-b_{\tau }^{*}+r_{ij}-\nabla h{\left(t_{ij},X_{i};\tilde{\phi }\right)}^{⊤}u/\sqrt{m}\}\\ & =\sum_{i=1}^{n}\sum_{j=1}^{m_{i}}\rho_{\tau }\{\epsilon_{ij}-b_{\tau }^{*}+r_{ij}-\zeta_{ij}\}, \end{array}$$

where $\zeta_{ij}=\nabla h{\left(t_{ij},X_{i};\tilde{\phi }\right)}^{⊤}u/\sqrt{m}$ and $\tilde{\phi }$ is between ϕ* and $\phi^{*}+u/\sqrt{m}$. Define

$$\begin{array}{cc} \Delta H & =H\left(\phi^{*}+u/\sqrt{m}\right)-H\left(\phi^{*}\right)\\ & =\sum_{i=1}^{n}\sum_{j=1}^{m_{i}}\rho_{\tau }(\epsilon_{ij}-b_{\tau }^{*}+r_{ij}-\zeta_{ij})-\sum_{i=1}^{n}\sum_{j=1}^{m_{i}}\rho_{\tau }(\epsilon_{ij}-b_{\tau }^{*}+r_{ij}). \end{array}$$

By the identity of [27],

$$\begin{array}{c} \left|r-s|-|r\right|=-s\left\{I\left(r>0\right)-I\left(r<0\right)\right\}+2\int_{0}^{s}\left\{I\left(r\leq x\right)-I\left(r\leq 0\right)\right\}dx. \end{array}$$

Hence, it can be obtained that

$$\begin{array}{c} \rho_{\tau }\left(r-s\right)-\rho_{\tau }\left(r\right)=s\left\{I\left(r<0\right)-\tau \right\}+\int_{0}^{s}\left\{I\left(r\leq x\right)-I\left(r\leq 0\right)\right\}dx. \end{array}$$

Thus, Δ*H* can be denoted as Δ*H* = Δ*H*_1_ + Δ*H*_2_, with

$$\begin{array}{cc} & \Delta H_{1}=\sum_{i=1}^{n}\sum_{j=1}^{m_{i}}\zeta_{ij}\left\{I\left(\epsilon_{ij}<b_{\tau }^{*}+r_{ij}\right)-\tau \right\},\\ & \Delta H_{2}=\sum_{i=1}^{n}\sum_{j=1}^{m_{i}}\int_{0}^{\zeta_{ij}}\left\{I\left(\epsilon_{ij}\leq x+r_{ij}+b_{\tau }^{*}\right)-I\left(\epsilon_{ij}\leq r_{ij}+b_{\tau }^{*}\right)\right\}dx. \end{array}$$

By calculating the expectation and variance of Δ*H*_2_,

$$\begin{array}{cc} E(\Delta H_{2}) & =E[\sum_{i=1}^{n}\sum_{j=1}^{m_{i}}\int_{0}^{\zeta_{ij}}\left\{I\left(\epsilon_{ij}\leq x+r_{ij}+b_{\tau }^{*}\right)-I\left(\epsilon_{ij}\leq r_{ij}+b_{\tau }^{*}\right)\right\}dx]\\ & =\sum_{i=1}^{n}\sum_{j=1}^{m_{i}}E[\int_{0}^{\zeta_{ij}}\{I\left(\epsilon_{ij}\leq x+r_{ij}+b_{\tau }^{*}\right)-I\left(\epsilon_{ij}\leq r_{ij}+b_{\tau }^{*}\right)\}dx]\\ & =\sum_{i=1}^{n}\sum_{j=1}^{m_{i}}\int_{0}^{\zeta_{ij}}\left\{G\left(x+r_{ij}+b_{\tau }^{*}\right)-G\left(r_{ij}+b_{\tau }^{*}\right)\right\}dx\\ & =\sum_{i=1}^{n}\sum_{j=1}^{m_{i}}\int_{0}^{\zeta_{ij}}\left\{G\left(x+r_{ij}+b_{\tau }^{*}\right)-G\left(r_{ij}+b_{\tau }^{*}\right)\right\}dx\\ & =\sum_{i=1}^{n}\sum_{j=1}^{m_{i}}\int_{0}^{\zeta_{ij}}xg\left(r_{ij}+b_{\tau }^{*}\right)dx+o_{p}\left(1\right)\\ & =\sum_{i=1}^{n}\sum_{j=1}^{m_{i}}\frac{\zeta_{ij}^{2}}{2}g\left(r_{ij}+b_{\tau }^{*}\right)+o_{p}\left(1\right)\\ & =\sum_{i=1}^{n}\sum_{j=1}^{m_{i}}\frac{\zeta_{ij}^{2}}{2}g\left(b_{\tau }^{*}\right)+o_{p}\left(1\right). \end{array}$$

By condition (C5), *E*[{∇*h*(*t*_*ij*_, *X*_*i*_;ϕ*)}^⊗2^] = Γ, we can have

$$\begin{array}{cc} E(\Delta H_{2}) & =\frac{g(b_{\tau }^{*})}{2m}\sum_{i=1}^{n}\sum_{j=1}^{m_{i}}u^{⊤}\nabla h\left(t_{ij},X_{i};\tilde{\phi }\right)\nabla h{\left(t_{ij},X_{i};\tilde{\phi }\right)}^{⊤}u\\ & =\frac{g(b_{\tau }^{*})}{2}u^{⊤}\sum_{i=1}^{n}\sum_{j=1}^{m_{i}}\frac{\nabla h\left(t_{ij},X_{i};\phi^{*}\right)\nabla h{\left(t_{ij},X_{i};\phi^{*}\right)}^{⊤}}{m}u+o_{p}\left(1\right)\\ & =\frac{g(b_{\tau }^{*})}{2}u^{⊤}\Gamma u+o_{p}\left(1\right). \end{array}$$

Next, we calculate the variance of Δ*H*_2_,

$$\begin{array}{cc} Var(\Delta H_{2}) & =Var[\sum_{i=1}^{n}\sum_{j=1}^{m_{i}}\int_{0}^{\zeta_{ij}}\left\{I\left(\varepsilon_{ij}\leq x+r_{ij}+b_{\tau }^{*}\right)-I\left(\epsilon_{ij}\leq r_{ij}+b_{\tau }^{*}\right)\right\}dx]\\ & \leq \sum_{i=1}^{n}\sum_{j=1}^{m_{i}}E{\left[\int_{0}^{\zeta_{ij}}\left\{I\left(\epsilon_{ij}\leq x+r_{ij}+b_{\tau }^{*}\right)-I\left(\epsilon_{ij}\leq r_{ij}+b_{\tau }^{*}\right)\right\}dx\right]}^{2}\\ & \leq \sum_{i=1}^{n}\sum_{j=1}^{m_{i}}\int_{0}^{|\zeta_{ij}|}\int_{0}^{|\zeta_{ij}|}\left\{G(\right|\zeta_{ij}|+r_{ij}+b_{\tau }^{*})-G\left(r_{ij}+b_{\tau }^{*}\right)\}dx_{1}dx_{2}=o_{p}\left(1\right). \end{array}$$

Hence, we can have $\Delta H_{2}=\left(1/2\right)g\left(b_{\tau }^{*}\right)u^{⊤}\Gamma u+o_{p}\left(1\right)$. Before discussing Δ*H*_1_, we first define $\kappa =\frac{1}{\sqrt{m}}\sum_{i=1}^{n}\sum_{j=1}^{m_{i}}\nabla h\left(t_{ij},X_{i};\phi^{*}\right)\left\{I\left({∊}_{ij}<b_{\tau }^{*}\right)-\tau \right\}$.

Then, we have *E*(*κ*) = 0 and *Var*(*κ*) = *τ*(1− *τ*)Γ. By the Cramer-Wald Theorem and the Central Limit Theorem, we can have that *κ* →_*d*_ *N*{0, *τ*(1 − *τ*)Γ}.

Next, we define

$$\begin{array}{c} \kappa_{1}=\frac{1}{\sqrt{m}}\sum_{i=1}^{n}\sum_{j=1}^{m_{i}}\nabla h\left(t_{ij},X_{i};\phi^{*}\right)\left\{I\left(\epsilon_{ij}<b_{\tau }^{*}+r_{ij}\right)-\tau \right\}, \end{array}$$

so that $\Delta H_{1}=\kappa_{1}^{⊤}\eta +o_{p}\left(1\right)$. By simple calculation, we have

$$\begin{array}{cc} & Var\left(\kappa_{1}-\kappa \right)=\frac{1}{m}Var[\sum_{i=1}^{n}\sum_{j=1}^{m_{i}}\nabla h\left(t_{ij},X_{i};\phi^{*}\right)\left\{I\left(\epsilon_{ij}<b_{\tau }^{*}+r_{ij}\right)-I\left(\epsilon_{ij}<b_{\tau }^{*}\right)\right\}]\\ & =\frac{1}{m}\sum_{i=1}^{n}\sum_{j=1}^{m_{i}}\nabla h\left(t_{ij},X_{i};\phi^{*}\right){\left\{\nabla h(t_{ij},X_{i};\phi^{*})\right\}}^{⊤}Var\left\{I\left(\epsilon_{ij}<b_{\tau }^{*}+r_{ij}\right)-I\left(\epsilon_{ij}<b_{\tau }^{*}\right)\right\}\\ & \leq \frac{1}{m}\sum_{i=1}^{n}\sum_{j=1}^{m_{i}}\nabla h\left(t_{ij},X_{i};\phi^{*}\right){\left\{\nabla h(t_{ij},X_{i};\phi^{*})\right\}}^{⊤}E|I\left(\epsilon_{ij}<b_{\tau }^{*}+r_{ij}\right)-I\left(\epsilon_{ij}<b_{\tau }^{*}\right)|\\ & \leq \frac{1}{m}\sum_{i=1}^{n}\sum_{j=1}^{m_{i}}\nabla h\left(t_{ij},X_{i};\phi^{*}\right){\left\{\nabla h\left(t_{ij},X_{i};\phi^{*}\right)\right\}}^{⊤}\{G\left(b_{\tau }^{*}+r_{ij}\right)-G\left(b_{\tau }^{*}\right)\}=o_{p}\left(1\right). \end{array}$$

Thus *κ*_1_ →_*p*_ *κ*. By Slutsky’s theorem, *κ*_1_ →_*d*_ *N*{0, *τ*(1 − *τ*)Γ}. Then, we can have that

$$\begin{array}{c} \Delta H=\frac{1}{2}g\left(b_{\tau }^{*}\right)u^{⊤}\Gamma u+\kappa_{1}^{⊤}u+o_{p}\left(1\right). \end{array}$$

By the epi-convergence results of [28], $\hat{u}\to_{d}-{g(b_{\tau }^{*})}^{-1}\Gamma^{-1}\kappa_{1}$. Finally, the asymptotic normality is proved $\hat{u}\to_{d}N\left\{0,g{\left(b_{\tau }^{*}\right)}^{-2}\tau \left(1-\tau \right)\Gamma^{-1}\right\}$.

Since ${∥{\hat{\eta }}_{k}\left(u\right)-\eta_{k}\left(u\right)∥}_{2}\leq {∥{\hat{\eta }}_{k}\left(u\right)-\gamma_{k}^{⊤}B\left(u\right)∥}_{2}+{∥\gamma_{k}^{⊤}B\left(u\right)-\eta_{k}\left(u\right)∥}_{2}$, we have

$$\begin{array}{cc} {∥{\hat{\eta }}_{k}\left(u\right)-\gamma_{k}^{⊤}B\left(u\right)∥}_{2} & ={\left\{E{\left({\hat{\gamma }}_{k}^{⊤}B\left(u\right)-\gamma_{k}^{⊤}B\left(u\right)\right)}^{2}\right\}}^{1/2}\\ & ={\left[E\{tr[{\left({\hat{\gamma }}_{k}-\gamma_{k}\right)}^{⊤}B\left(u\right)B{\left(u\right)}^{⊤}\left({\hat{\gamma }}_{k}-\gamma_{k}\right)]\}\right]}^{1/2}\\ & ={\left[tr\{E\left(B\left(u\right)B{\left(u\right)}^{⊤}\right)E\left({\hat{\gamma }}_{k}-\gamma_{k}\right){\left({\hat{\gamma }}_{k}-\gamma_{k}\right)}^{⊤}\}\right]}^{1/2}\\ & =O_{p}[{\left\{g{\left(b_{\tau }^{*}\right)}^{-2}\tau \left(1-\tau \right)\right\}}^{1/2}{\left(L/\sum_{i=1}^{n}m_{i}\right)}^{1/2}]=O_{p}\{{\left(L/m\right)}^{1/2}\}. \end{array}$$

By the Lemma 1, ${∥{\hat{\eta }}_{k}\left(u\right)-\eta_{k}\left(u\right)∥}_{2}=O_{p}\{{\left(L/m\right)}^{1/2}\},k=1,\ldots ,p+1$. Thus, we can get

$$\begin{array}{c} {∥\;\text{log}{\hat{\lambda }}_{0}\left(u\right)-\;\text{log}\lambda_{0}\left(u\right)∥}_{2}=O_{p}\{{\left(L/m\right)}^{1/2}\}, \end{array}$$

and

$$\begin{array}{c} {∥{\hat{\beta }}_{k}\left(u\right)-\beta_{k}\left(u\right)∥}_{2}=O_{p}\{{\left(L/m\right)}^{1/2}\},k=1,\cdots ,p. \end{array}$$
